# Longitudinal and Transverse Relaxivity Analysis of Native Ferritin and Magnetoferritin at 7 T MRI

**DOI:** 10.3390/ijms22168487

**Published:** 2021-08-06

**Authors:** Oliver Strbak, Lucia Balejcikova, Martina Kmetova, Jan Gombos, Jozef Kovac, Dusan Dobrota, Peter Kopcansky

**Affiliations:** 1 Biomedical Center Martin, Jessenius Faculty of Medicine in Martin, Comenius University in Bratislava, Mala Hora 4, 036 01 Martin, Slovakia; 2 Institute of Hydrology, Slovak Academy of Sciences, Dubravska Cesta 9, 841 04 Bratislava, Slovakia; balejcikova@uh.savba.sk; 3 Department of Medical Biochemistry, Jessenius Faculty of Medicine in Martin, Comenius University in Bratislava, Mala Hora 4, 036 01 Martin, Slovakia; martinamihalikova09@gmail.com (M.K.); gomboss.jan@gmail.com (J.G.); dusan.dobrota@uniba.sk (D.D.); 4 Institute of Experimental Physics, Slovak Academy of Sciences, Watsonova 47, 040 01 Kosice, Slovakia; jkovac@saske.sk (J.K.); kopcan@saske.sk (P.K.)

**Keywords:** native ferritin, magnetoferritin, loading factor, MRI, relaxation, longitudinal and transverse relaxivity

## Abstract

Magnetite mineralization in human tissue is associated with various pathological processes, especially neurodegenerative disorders. Ferritin’s mineral core is believed to be a precursor of magnetite mineralization. Magnetoferritin (MF) was prepared with different iron loading factors (*LFs*) as a model system for pathological ferritin to analyze its MRI relaxivity properties compared to those of native ferritin (NF). The results revealed that MF differs statistically significantly from NF, with the same *LF*, for all studied relaxation parameters at 7 T: *r*_1_, *r*_2_, *r*_2_*, *r*_2_*/r*_1_, *r*_2_*/r_1_. Distinguishability of MF from NF may be useful in non-invasive MRI diagnosis of pathological processes associated with iron accumulation and magnetite mineralization (e.g., neurodegenerative disorders, cancer, and diseases of the heart, lung and liver). In addition, it was found that MF samples possess very strong correlation and MF’s relaxivity is linearly dependent on the *LF*, and the transverse and longitudinal ratios *r*_2_*/r*_1_ and *r*_2_*/r_1_ possess complementary information. This is useful in eliminating false-positive hypointensive artefacts and diagnosis of the different stages of pathology. These findings could contribute to the exploitation of MRI techniques in the non-invasive diagnosis of iron-related pathological processes in human tissue.

## 1. Introduction

Iron is an essential element for almost all living systems, including humans [1]. However, excess levels of biogenic iron are associated with a variety of human pathological processes, including inflammation [2], neurodegeneration [3], neuroinflammation [4], and even cancer [5]. Moreover, it is also connected with disorders of the liver [6], heart [7], and lung [8]. Iron accumulation in tissues and the formation of aggregates as nanosized iron oxide particles [3], especially magnetite [9,10], is often described for neurodegenerative disorders and is believed to be associated with disrupted iron homeostasis [11]. It is generally accepted that the toxicity of iron is a result of the ability of ferrous ions to produce hydroxyl radicals through the Fenton reaction [12]. This process is hazardous, resulting in hydrogen peroxide overproduction and damage to antioxidants, including iron scavengers, peroxidases, and iron storage proteins, such as ferritin [13], which can lead to cancer [14]. From these studies, together with recent work on reduction of the ferritin nucleus [15], it appears that iron accumulation and mineralization is at least an accomplice of pathology. However, at present, it is still not known whether the formation of iron oxide aggregates is the cause or the result of pathology.

It has been suggested that a precursor of iron accumulation and pathological mineralization is ferritin [16]. A ferritin particle is an iron storage molecule, composed of a protein envelope (12 nm) and a central cavity (8 nm) with a mineral core in the form of a crystalline ferrihydrite-like compound [17]. The primary role of ferritin in living systems is to remove toxic ferrous ions when they reach their critical cell concentration and to deposit them as non-toxic ferric ions for later usage by the organism. 

Surprisingly, the mineral core composition of ferritin is changed by pathology. In 2004, Quintana et al. showed, by using electron nanodiffraction and electron microscopy, that the brain-tissue ferritin mineral core of patients with Alzheimer’s disease (referred to as “pathological” ferritin) is structurally different from native (“physiological”) ferritin [16]. In physiological ferritin, the mineral core consists mainly of hexagonal ferrihydrite, hematite, and a smaller phase of magnetite. In contrast, the core of pathological ferritin consists mainly of cubic structures, such as magnetite and wüstite, and to a lesser extent ferrihydrite, but no hematite. These conclusions have been confirmed by Bossoni et al. [18]. By using muon spin rotation they showed that ferritin particles from healthy subjects contain ferrihydrite, but particles from Alzheimer’s disease patients have a crystalline phase with large magnetocrystalline anisotropy compatible with magnetite or maghemite. In the following text, for simplicity, we will describe the mineral core of NF as a ferrihydrite-like core and the mineral core of MF as a magnetite-like core. Due to the predominance of these mineral phases in these particles, we consider this to be an acceptable approximation.

From this perspective, pathological brain-tissue ferritin can be described as magnetoferritin (MF), which is composed of apoferritin and an artificially added phase of magnetite or maghemite [19]. MF thus represents a suitable model system of pathological brain-tissue ferritin that enables the study of the relaxation properties of pathological ferritin in different concentrations and loading factors (*LFs*)—number of iron atoms per protein [20]. This is required for the correct interpretation of in vivo data, including endogenous iron oxides in different phases and concentrations. The effect of magnetite-containing minerals in pathological ferritin (MF) on longitudinal and transverse relaxivity is expected to be 100–1000 times larger compared to the impact of native ferritin (NF) [21]. This follows from the significantly larger magnetic moment of MF (≈13,000 µB [22]) compared to an NF core (≈300 µB [23]). This should allow differentiation between pathological ferritin (with a magnetite-like mineral core) and physiological ferritin (with a ferrihydrite-like mineral core).

Although this theory has not been confirmed directly by nuclear magnetic resonance (NMR) [24], clinical studies suggest a correlation between hypointensive artefacts in T_2_ and T_2_* weighted images and the presence of neurodegeneration [25,26]. This indirectly supports the idea of magnetite-like relaxation in pathological tissue because, at present, it cannot be explained by any other mechanism. The reason for the discrepancy with the NMR study [24] may be that they did not include magnetite particles independent of the protein envelope in their experiments, instead only considering particles with a maximum size of 7 nm. However, in Alzheimer’s disease tissue, magnetite particles with an average size of 33 ± 15 nm and with a maximum diameter up to 200 nm were found [9]. Thus, pathological ferritin could look like decayed MF, where the mineral core serves as a template for further crystal growth, or aggregation of magnetite nuclei due to their dipole interactions. In a recent study, it was confirmed that MF could be distinguished from NF, both in low-field (0.2 T) and high-field (4.7 T) magnetic resonance contrast imaging [20], by comparing the relative contrast and relaxation time values. In the present study, a quantitative and qualitative analysis of MF-induced relaxivity changes at 7 T magnetic resonance imaging will be provided. The main goal is to find out whether it is possible to distinguish in vitro NF from MF as a model system of pathological ferritin by comparison of the relaxivity values. This should have a direct impact on medical applications. The next goal is to determine the correlation and causality of the relaxivity of ferritin samples with respect to the *LF*, which would enable the diagnosis of the stage of the disease. 

## 2. Results and Discussion

The basic physicochemical characteristics (*LF*, *D_<HYDR>_*, *ζ potential*, polydispersity index—*PDI*) of the NF (horse spleen ferritin) and MF samples are shown in Table 1. The *LF*, *D_<HYDR>_* and *ζ potential*, including magnetization curves, of the mineral core reduction of NF and MF, induced by vitamins B_2_ and C, have recently been published by this group [15]. Summarizing this earlier work, the hydrodynamic diameter *D_<HYDR>_*, determined by dynamic light scattering (DLS), has higher values of MF than NF, probably due to the aggregation of MFs as a consequence of their increased dipole–dipole interactions. This is supported by the observation that NF and MF3 possess almost the same *LF* but differ significantly in *D_<HYDR>_* and *PDI*. This suggests that the altered mineral core composition is a key factor in the MF size distribution. The *PDI* points to a broader particle size distribution in all MF samples, which was our intention, as pathological processes in vivo produce a similar formation of aggregates and protein degradation [9,10]. The highest PDI value is for the MF3 sample, which also possesses the highest *LF*. Thus, the *LF* (amount of loaded iron) seems to be a determining factor for the *D_<HYDR>_* and *PDI* in MF samples. This is supported by the visible time-dependent sedimentation of agglomerates and *ζ potential* decrease in MF samples. *ζ potential* refers to the negative total charge of proteins that provide electrostatic repulsion between particles and comes from the negatively charged amino-acid residues on the protein surface. The value of the *ζ potential* is related to the viscosity of the aqueous environment and is probably influenced by the *LF* and iron core type. However, the higher *ζ potential* and *PDI* in MF does not mean the samples are unstable. The presence of some monodisperse particles is not excluded; this was demonstrated by the pH variation (changing to lower values indicates the presence of core-shell structures) and by Small-Angle X-ray Scattering (SAXS) [27]. MF samples were prepared to mimic magnetite mineralization and aggregation, which have been found in various pathological processes. To achieve magnetite crystallization in the middle of the synthesis of MF, 2 M NaOH solution was added [28] to prevent the lepidocrocite crystallization that was observed in samples prepared by the standard MF synthetic procedure [29]. The change in pH increased the magnetization, but it also probably partially destroyed the protein coat, as proved in a recent study [27]. Uncoated mineral cores can thus interact with each other forming larger aggregates which were observed, for example, in neurodegenerative diseases [9,10]. The magnetite particles thus prepared formed an ideal model system to study the MRI relaxation properties of physiological and pathological ferritin. 

To determine the relaxivity, NF and MF samples with different *LFs* (Table 1) were measured with three different MRI relaxation time mapping protocols: (i) *T*_1_ mapping RARE protocol (Figure S1); (ii) *T*_2_ mapping MSME protocol (Figure S2); (iii) *T*_2_* mapping MGE protocol (Figure S3). To determine the correlation and causality of different *LFs* (*D_<HYDR>_* and *ζ potential*) on basic MRI parameters in MF and NF the relative contrast (*RC)* (Figure S4), relaxation time *T* (Figure S5) and relaxation rate *R* (Figure S6) of both NF and MF samples were determined. For all determined values of the relaxation time, the “observed” relaxation time *T*_2_* is less than the “true” relaxation time *T*_2_, which is to be expected [30]. The reason for this difference is the different physical properties of the *T*_2_ and *T*_2_* weighted sequences. *T*_2_ correlates with the representation of the slow-moving fraction of water molecules. The more slow-moving water-binding macromolecules that are present in the sample, the lower the *T*_2_ value. In addition, the *T*_2_* value is also affected by the inhomogeneities of the static magnetic field that are caused primarily by the presence of paramagnetic substances in the sample. 

**Figure 1:**
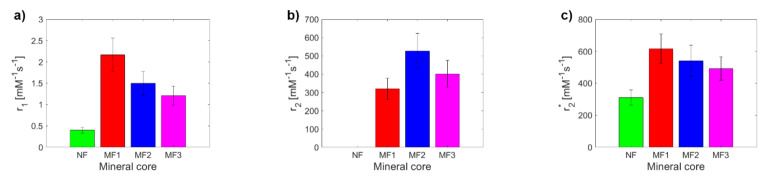
Comparison of relaxivity values of NF and MF samples: (**a**) longitudinal relaxivity *r*_1_*;* (**b**) transverse relaxivity *r*_2_; (**c**) transverse relaxivity *r*_2*_.

By fitting the longitudinal and transverse relaxation rates *R* (Figure S7), the relaxivity *r* was determined (Figure 1). In all cases (different *LF* values), it was possible to differentiate the relaxivity of NF from MF, as well as the different *LF* of MF. The difference between NF and MF is the biggest for *r*_2_. The transverse relaxivity *r*_2_ of NF is of the order of 1–10 mM^−1^s^−1^ at a typical magnetic field strength 1–10 T [31], which is consistent with these measurements (1.27 ± 0.23 mM^−1^s^−1^). In comparison with the findings of Jordan et al., whose MF preparation includes the incorporation of iron oxide molecules into the protein core via a step-wise Fe(II) chloride addition to the protein solution under low O_2_ conditions [32], their per-iron transverse relaxivity values *r*_2_ obtained by Spin echo sequencing are as follows: NF: *r*_2_ = 4 mM^−1^s^−1^ (this study: 1.27 ± 0.23 mM^−1^s^−1^), MF: *r*_2_ = 130 mM^−1^s^−1^ (this study: 402 ± 72 mM^−1^s^−1^). The per-iron longitudinal relaxivity *r*_1_ is 0.07 mM^−1^s^−1^ compared to the values found in this study, which are from 1.21 ± 0.22 to 2.17 ± 0.39 mM^−1^s^−1^ for different *LF* values. This difference is probably due to a different concentration range for the relaxivity calculation ([32]: 0.00–0.05 mM of iron, this study: 0.00–0.20 mM of iron) and different *LF* values, which they do not report. In general, typical superparamagnetic agents, composed of paramagnetic iron oxide ions, have low longitudinal relaxivity *r*_1_ (≈1 mM^−1^s^−1^) but high transverse relaxivity *r*_2_ (≈100 mM^−1^s^−1^) [32], which is in agreement with the findings of this study for the MF samples (Table 1).

NF has lower values than MF for *r*_1_ and *r*_2_*. For *r*_1_ and *r*_2_*, a decrease in MF relaxivity with increasing *LF* was observed, while in the case of *r*_2,_ it was the opposite. From a theoretical point of view, the increase in *LF* should be accompanied by an increase in relaxivity, as is seen in the case of “true” relaxivity *r*_2_. The reason for the opposite course of “observed” relaxivity *r*_2*_ (a decrease in *r* value with an increase in *LF*) is unknown and is outside the scope of this publication. The higher relaxivity of the MF2 sample compared to the MF3 sample (Figure 1b) could be caused by the higher sedimentation/aggregation of MFs in MF2, since the *T*_2_ mapping protocols require a much larger time for measurement, in comparison with *T*_2_* protocols. In any case, using all three observed values of the relaxivity (*r*_1_, *r*_2_, *r*_2*_), it is possible to differentiate NF from MF as a model system of pathological ferritin. 

**Figure 2:**
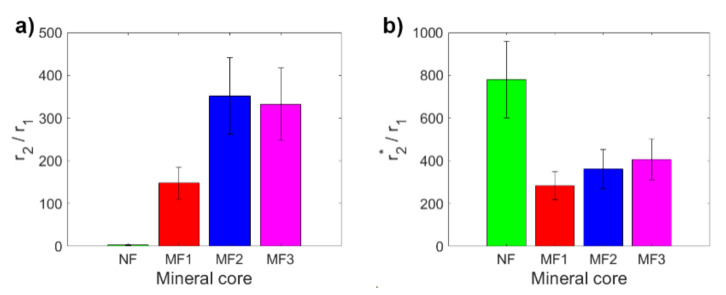
Comparison of relaxivity ratios of NF and MF samples: (**a**) *r*_2_*/r*_1_; (**b**) *r*_2*_/r_1_.

A key tool for comparing the MRI contrast of different samples is the ratio of transverse and longitudinal relaxivity *r*_2_*/r*_1_ (*r*_2*_/r_1_). Figure 2 shows a comparison of these ratios for the studied NF and MF samples. The *r*_2_*/r*_1_ ratio is for MF samples in a range from 148 ± 38 to 352 ± 90 (Figure 2a), which is lower than the findings of Jordan et al. (*r*_2_*/r*_1_ = 1114) [32]. In the case of the *r*_2*_/r_1_ ratio, it is from 284 ± 66 to 407 ± 96 (Figure 2b), which is higher than the *r*_2_*/r*_1_ ratio, as predicted. Except for MF2, the values of the ratios for MF show the expected pattern; they increase with an increase in *LF*. Of particular interest is the high value of the *r*_2*_/r_1_ ratio for NF, which is due to the very high value of the NF transverse relaxivity *r*_2*_ and the low NF longitudinal relaxivity *r*_1_. This peculiarity is probably associated with the change of magnetic properties in nanosized ferrihydrite to superparamagnetic properties, although bulk ferrihydrite exhibits antiferromagnetic behavior [33]. Paramagnetic compounds affect the homogeneity of the magnetic field; the *T*_2_* relaxation time is very sensitive to this. However, as in the case of individual relaxivities and also for their ratios (*r*_2_*/r*_1_, *r*_2*_/r_1_), it is possible to clearly distinguish MF from NF, which is an important result for possible applications.

Based on the relaxivity findings, it was possible to determine the relationship between the different *LF* of NF and MF cores by calculating the correlation coefficients for all defined MRI parameters (Table 2, Figures S8–S10). Since the samples were prepared with increasing concentration gradients of iron oxide they vary from a normal distribution (analyzed using the Kolmogorov–Smirnov test at the 5% significance level, unpublished data). The Spearman method was used to calculate the correlation coefficients [34]. For interpreting correlation coefficient data, the following conventional approach was used: negligible correlation (0.00–0.10), weak correlation (0.10–0.39), moderate correlation (0.40–0.69), strong correlation (0.70–0.89) and very strong correlation (0.90–1.00) [34]. It was found that all calculated correlation coefficients are positive (Table 2, Figures S8–S10). Except for two values (strong correlation in MF2-MF3 *T*_2_ and *R*_2_), all MF samples show a very strong correlation for all observed MRI parameters. Based on the magnetite structure of the MF mineral core and its increasing *LF*, this is the expected result and proves the credibility of the MF samples preparation methodology.

The situation is more diverse in the case of the correlation between NF and MF, from very strong correlation (*RC T*_2_*w*), through strong correlation (*RC T*_2_*w, *T*_1_, *R*_1_) to moderate correlation (*RC T*_1_*w*, *T*_2_*, *R*_2*_). However, in the case of transverse relaxation time *T*_2_ and relaxation rate *R*_2_, only a weak correlation was observed for NF-MF2, which is probably associated with the sedimentation/aggregation of MFs in MF2, as discussed above. 

**Figure 3:**
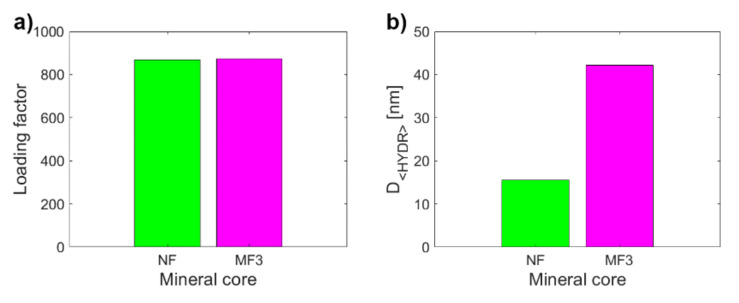

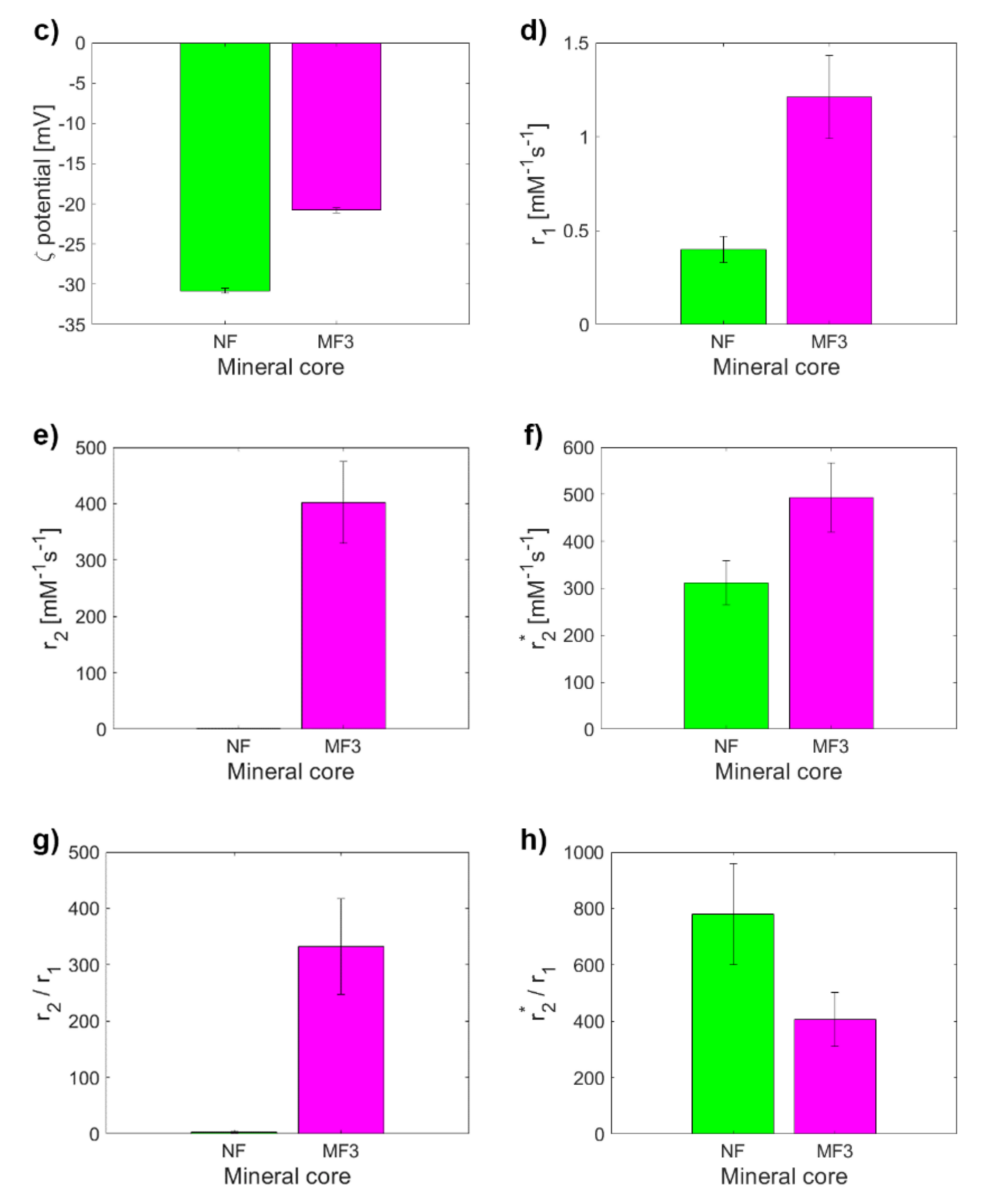
Comparison of the observed variables for NF and MF3, which have almost the same *LF* (868.00 vs. 873.00): (**a**) *LF*; (**b**) *D_<**HYDR**>_*; (**c**) ***ζ*** *potential*; (**d**) longitudinal relaxivity *r*_1_; (**e**) transverse relaxivity *r*_2_; (**f**) transverse relaxivity *r*_2*_; (**g**) relaxivity ratio *r*_2_*/r*_1_; (**h**) relaxivity ratio *r*_2*_/r_1_.

To assess the clinical application of these results it is essential to determine to what extent it is possible to distinguish the physiological mineral core of ferritin (ferrihydrite-like) from the pathological mineral core (magnetite-like). Therefore, the MRI relaxivities of NF and MF3, which have almost the same *LF* (Figure 3a) but different hydrodynamic diameters (Figure 3b) and *ζ potential* (Figure 3c), were compared. However, both samples also differ statistically significantly in all of the relaxivities compared: *r*_1_, *r*_2_, *r*_2*_, *r*_2_*/r*_1_, *r*_2*_/r_1_ (Figure 3d–h). The largest difference (an almost 320-fold increase) between NF and MF3 is observed for transverse relaxivity *r*_2_ (Figure 3e), followed by relaxivity ratios *r*_2_*/r*_1_ (an almost 105-fold increase, Figure 3g). Transverse relaxivity *r*_2_ values for all MF samples (Table 1) are higher than the transverse relaxivity of commercially used iron oxide-based contrast agents: Feridex r_2_ = 120 mM^−1^s^−1^, Resovist r_2_ = 186 mM^−1^s^−1^ and Combidex r_2_ = 65 mM^−1^ s^−1^ [35]. The transverse and longitudinal relaxivity ratio *r*_2_*/r*_1_ is a key feature when comparing the MRI contrast efficacy of the *T*_2_ comparison compounds, such as iron oxide-based nanoparticles. The higher the ratio, the better the contrast efficacy and the visibility of the agent [36]. In this case, the ratio *r*_2_*/r*_1_ is 3.18 ± 0.80 mM^−1^s^−1^ for NF compared to *r*_2_*/r*_1_ = 332 ± 85 mM^−1^s^−1^ for MF3, which is even higher than the value for developed magnetite-based contrast agents with the same concentration and in the same magnetic field [37]. Thus, pathologically mineralized magnetite in ferritins should behave like an intrinsic, biogenic iron oxide-based contrast agent. In the case of longitudinal relaxivity *r*_1_, a threefold increase in favor of the magnetite mineral core in the MF3 sample was observed. This is not surprising since iron oxides shorten both *T*_1_ and *T*_2_ relaxation times, although for *T*_2_ it is much more pronounced, as was observed in this study (Table 1). The “observed” relaxivity *r*_2*_ in the case of both NF and MF3 is higher than the “true” relaxivity *r*_2_, which agrees with the theory. However, even though ferrihydrite is antiferromagnetic [38] and should have no effect on the main magnetic field, a dramatic increase in the *r*_2*_ value for NF in comparison with *r*_2_ was observed. This was probably caused by a change in magnetic properties of nanosized ferrihydrite to superparamagnetic behavior [33], which has a clear effect on the homogeneity of the magnetic field. An inverted result for the relaxivity ratios *r*_2*_/r_1_ compared to *r*_2_*/r*_1_ could be used to exclude other artefacts (e.g., tissue calcification) in in vivo diagnostics of pathological iron mineralization from the physiological state.

**Figure 4:**
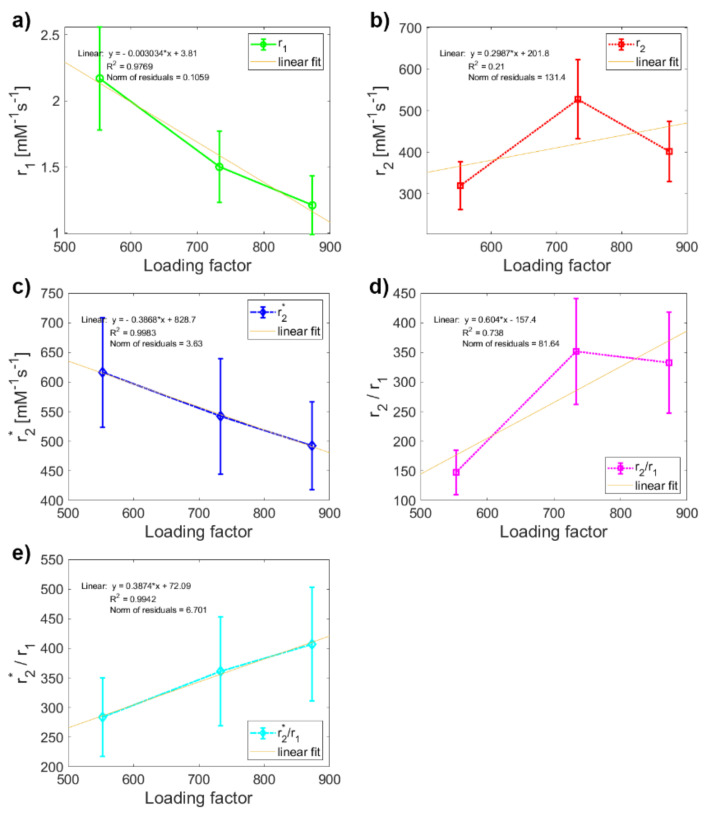
Linear regression analysis of the dependence of relaxivity on *LF*: comparison of variables Figure 3. samples: (**a**) longitudinal relaxivity *r*_1_; (**b**) transverse relaxivity *r*_2_; (**c**) transverse relaxivity *r*_2*_; (**d**) relaxivity ratio *r*_2_*/r*_1_*;* (**e**) relaxivity ratio *r*_2_*/r_1_.

In the last step, regression analysis was used to reveal how the *LF* affects the relaxivity of MF mineral core and whether it would be possible to use this relationship to predict the rate of pathological magnetite-like iron accumulation and mineralization in tissue. Linear regression analysis showed a clear linear dependence (R^2^ ≈ 1) of MF sample relaxivity on the value of *LF*, except for transverse relaxivity *r*_2_ (Figure 4). As discussed above, since this anomaly does not occur in the case of longitudinal relaxivity *r*_1_ and transverse relaxivity *r*_2*_, we believe that it is caused only by higher sedimentation of MFs in the MF2 sample during the longer data acquisition in *T*_2_ weighted pulse sequence compared to *T*_1_ and *T*_2_*-weighted protocols. Therefore, it affects only the “true” relaxation time *T*_2_ and not the “observed” relaxation time *T*_2_* and longitudinal time *T*_1_. The theoretical value of the MF2 transverse relaxivity *r*_2_ obtained by extrapolation is 366 mM^−1^s^−1^, compared to the value of 528 mM^−1^s^−1^ obtained in this study. However, this inaccuracy does not affect the conclusion that the relaxivity of MF is linearly dependent on the *LF* and it would therefore be possible to determine the amount of accumulated pathological magnetite-like iron in tissue from relaxivity values.

## 3. Materials and Methods

### 3.1. Chemicals

Ammonium ferrous sulfate hexahydrate ((NH_4_)_2_Fe(SO_4_)_2_·6H_2_O), equine spleen apoferritin in 0.15 M NaCl, ethanol (C_2_H_5s_OH), horse spleen ferritin in 0.15 M NaCl, hydrogen peroxide (H_2_O_2_), 3-[(1,1-dimethyl-2-hydroxyethyl)amino]-2-hydroxypropanesulfonic acid (AMPSO), sodium hydroxide (NaOH), and trimethylamine N-oxide (Me_3_NO) were obtained from SIGMA-Aldrich (Saint-Louis, MO, USA); Coomassie brilliant blue from Fluka (Buchs, Switzerland); hydrochloric acid (HCl) from ITES (Vranov nad Toplou, Slovakia); potassium thiocyanate (KSCN) from Slavus (Bratislava, Slovakia); phosphoric acid (H_3_PO_4_) from Centralchem (Bratislava, Slovakia), and demineralized water.

### 3.2. Synthesis of Magnetoferritin

We used horse spleen ferritin as a model system of native ferritin. MF was prepared by the procedure described elsewhere [20]. Ferrous ions were added to the empty protein shell of a native apoferritin (NA) solution with a protein concentration of ≈3 mg/mL. First, the apoferritin solution was added to a 0.05 M AMPSO buffer with the pH adjusted to 8.6 using 2 M NaOH solution, utilizing a pH meter (Mettler Toledo SevenEasy S20-KS) and a pH electrode (Mettler Toledo Inlab^®^Science Pro). The reaction bottle containing apoferritin solution was hermetically closed. The demineralized water for the solutions’ preparation was deaerated using nitrogen for 1 h to provide anaerobic conditions and controlled oxidation. For the ferrous ion source, we used a 0.1 M solution of Mohr’s salt ((NH_4_)_2_Fe(SO_4_)_2_·6H_2_O) and a stoichiometric amount of a 0.07 M solution of trimethylamine N-oxide. The synthesis was carried out by the gradual addition of the reactants in 10 steps over 100 min using syringes at 65 °C under constant stirring via a magnetic stirrer with heating (IKA C-MAG HS 7). MF was prepared with three different *LFs*: 553, 733, and 872. The *LF* of NF was 868.

### 3.3. Quantitative Determination of the Loading Factor

We quantitatively analyzed the *LF* by Ultraviolet–Visible (UV-VIS) spectrophotometer (SPECORD 40, Analytik Jena, Jena, Germany) at 25 °C with a precision of approximately 1%. The mass concentration of iron atoms $c_{m}^{Fe}$ was determined after oxidation of Fe^2+^ to Fe^3+^ ions with 3% H_2_O_2_ in an acid medium of 35% HCl at 50 °C for 30 min. After the addition of 1 M KSCN, a red thiocyanate complex of Fe[Fe(SCN)_6_] was produced and its absorbance was measured at 450 nm. From the calibration curve, using the regression equation, the corresponding mass concentration of iron atoms was calculated. The standard Bradford method was used to determine the mass concentration of NA, $c_{m}^{NA}$. The absorbance of the blue protein complex of the Bradford agent was detected at 595 nm after 5 min incubation at 25 °C. From the calculated ratio of $c_{m}^{Fe}$a:$c_{m}^{NA}$ in a given volume of the sample using the known molecular weights of apoferritin and iron, respectively, the *LF* of MF was calculated according to the equation:

(1) $$LF=\frac{c_{m}^{Fe}\cdot M_{NA}}{c_{m}^{NA}\cdot M_{Fe}}$$

### 3.4. Measurement of the Hydrodynamic Diameter

The hydrodynamic diameter *D_<HYDR>_*of the samples in aqueous solutions was measured using a Zetasizer NanoZS 3600 (Malvern Instruments, Malvern, UK) at 25 °C based on the principle of DLS, also known as photon correlation spectroscopy or quasi-elastic light scattering. DLS analyzes the intensity fluctuations of the scattered light from particles performing Brownian motion and measures the rate of the particles’ diffusion in the solution, which is related to their size as described by the Stokes–Einstein equation:

(2) $$D_{T}=\frac{k\cdot T}{6\cdot \pi \cdot \eta \cdot R_{h}}$$

where *D_T_* is the diffusion coefficient, *k* is the Boltzman constant, *T* is the temperature, *η* is the solvent viscosity and *R_h_* is the Stokes (or hydrodynamic) radius of the spherical particles (in nm).

The final *D_<HYDR>_*values were obtained from triplicate measurement of aqueous protein samples in disposable polystyrene cuvettes using the protein data analysis mode. *D_<HYDR>_* was calculated by averaging sizes, displayed as the maximum of the size distribution curve from the number function (PSD). In addition, the size distribution and the *PDI* were evaluated and compared. The *PDI* ranges from 0 for uniformly sized particles to 1 for highly polydisperse particles. 

### 3.5. Magnetometry

The magnetic properties of the iron oxide mineral cores inside the protein shell were studied using a SQUID magnetometer MPMS 5XL (Quantum Design, San Diego, CA, USA). The magnetization curves of the MF samples were measured at 290 K and magnetic strength up to 5 T.

### 3.6. Magnetic Resonance Imaging

The MRI measurements were performed using a 7 T BioSpec Bruker system. Three different protocols were used for *T*_1_, *T*_2_ and *T*_2_* parametric mapping:

- *T*_1_ mapping—Rapid Acquisition with Refocused Echoes (RARE) pulse sequence, with repetition time *T_R_* = 5500, 3000, 1500, 800, 400 and 200 ms, and echo time *T_E_* = 7 ms.
- *T*_2_ mapping—Multi-Slice Multi-Echo (MSME) pulse sequence, with repetition time *T_R_* = 2000 ms, starting echo time *T_E_* = 8 ms, spacing = 8 ms, and 25 images.
- *T*_2_* mapping—Multi Gradient Echo (MGE) pulse sequence, with repetition time *T_R_* = 1200 ms, starting echo time *T_E_* = 5.1 ms, spacing = 5 ms, and 10 images.

The concentration gradient (2.5 × 10^−3^–0.02 mg/mL) of ferrihydrite in ferritin and magnetite in MF was prepared to perform relaxivity measurements. First, the signal intensity values (*I*_0_—without iron oxide core and *I*—with iron oxide core) were acquired and evaluated as the relative contrast (*RC*). The *RC* of iron oxides that are characterized as a negative contrast agent (*I*_0_ > *I*) is defined as follows:

*RC* = (*I* − *I*_0_)/*I*_0_ (3)

where *I*_0_ is the signal intensity without iron oxide particles and *I* is the signal intensity with iron oxide particles.

Subsequently, the longitudinal and transverse relaxation times (*T*_1_, *T*_2_ and *T*_2_*) were determined by fitting with the following functions:

*M*(*t*) = *A* + *M*_0_ × (1 − *exp*(*t*/*T*_1_)) (4)

*y* = *A* + *C* × *exp*(−*t*/*T*_2_) (5)

where *M*_0_ is the equilibrium magnetization, *A* is the absolute bias, *T*_1_ is the longitudinal recovery time, *C* is the signal intensity and *T*_2_ is the transverse relaxation time. 

The value of *T*_2_ is only influenced by atomic molecular interactions, while the *T*_2_* value reflects atomic molecular interactions, as well as inhomogeneities in the main magnetic field (B_0_). Finally, the transverse and longitudinal relaxation rates (*R*_1_, *R*_2_ and *R*_2*_) and relaxivity (*r*_1_, *r*_2_, *r*_2*_) values were calculated and evaluated. The transverse relaxation rate (*R_n_*) is the inverse of the relaxation time (*T_n_*):

*R_n_* = 1/*T_n_* (*n* = 1 or 2) (6)

The change in *R_n_* characterizes the efficiency of the magnetic particle MRI contrast properties and is defined as the relaxivity of the particle (contrast agent):

*r_n_* = (*R_n_* − *R_n_*^0^)/*C_IO_* (*n* = 1 or 2) (7)

where *R_n_*^0^ is the relaxation rate in the absence of magnetic (iron oxide) particles, *R_n_* represents the relaxation rate in the presence of magnetic particles and *C_IO_* is the particle (iron oxide) concentration. The relaxivity value *r_n_* using a linear fit of the relaxation rate *R_n_* dependence on the molar concentration of iron in ferrihydrite and magnetite was determined.

A Paravision “Image Sequence Analysis” tool (Bruker, Billerica, MA, USA) and a Matlab R2021a software tool (Mathworks Inc., Natic, MA, USA) were used for MRI data processing and visualization.

### 3.7. Statistics

Basic statistics (mean, standard deviation, fitting parameters), including the Kolmogorov–Smirnov test for normality, the calculation of Spearman correlation coefficients and linear regression analysis, were performed using a Matlab R2021a software tool (Mathworks Inc., Natic, MA, USA).

## 4. Conclusions

The MRI relaxivity at 7 T in NF and MF with different *LF* was analyzed to show the differences in longitudinal and transverse relaxivity as a model system for pathological ferritin. The relaxivities *r*_1_, *r*_2_, and *r*_2*_ were obtained by comparing the relaxation rates (*R*_1_, *R*_2_, and *R*_2*_), which show that NF differs significantly from MF with various *LF* for all studied relaxation parameters: *r*_1_, *r*_2_, *r*_2*_, *r*_2_*/r*_1_, *r*_2*_/r_1_. This may be useful in non-invasive MRI diagnosis of the pathological processes associated with iron accumulation and magnetite mineralization in ferritin (e.g., neurodegenerative disorders, cancer, and diseases of the heart, lungs and liver). In addition, it was found that MF samples possess very strong correlation and MF relaxivity is linearly dependent on the *LF* for all the studied relaxation parameters (*r*_1_, *r*_2_, *r*_2*_, *r*_2_*/r*_1_, *r*_2*_/r_1_). The transverse and longitudinal ratios *r*_2_*/r*_1_ and *r*_2*_/r_1_, as basic MRI contrast variables, exhibit complementary information. This can be useful in eliminating false-positive hypointensive artefacts and the diagnosis of the stage of pathology (related to the stage of mineralization and *LF*). These findings contribute to the understanding of iron oxide accumulation in early non-invasive MRI diagnosis of the pathological processes related to disrupted iron homeostasis and magnetite mineralization in tissue.

## Figures and Tables

**Table 1:** The physico-chemical properties and relaxivity values of NF and MF: (i) loading factor (*LF);* (ii) hydrodynamic diameter (*D_<HYDR>_);* (iii) *ζ potential*; (iv) polydispersity index (*PDI*); (v) longitudinal relaxivity *r*_1_; (vi) transverse (“true”) relaxivity *r*_2_; (vii) transverse (“observed”) relaxivity *r*_2*_; (viii) relaxivity ratio *r*_2_*/r*_1_; (ix) relaxivity ratio *r*_2*_/r_1_.

	NF	MF1	MF2	MF3
*LF*	868.00 ± 0.03	553.00 ± 0.08	733.00 ± 0.42	873.00 ± 0.11
*D_<HYDR>_* (nm)	15.600 ± 0.002	46.000 ± 0.003	46.500 ± 0.056	42.200 ± 0.012
*ζ potential* (mV)	–30.8 ± 0.3	–29.3 ± 0.5	–26.9 ± 0.1	–20.8 ± 0.3
*PDI*	0.30 ± 0.02	0.40 ± 0.02	0.40 ± 0.03	0.60 ± 0.01
*r*_1_ (mM^−1^s^−1^)	0.40 ± 0.07	2.17 ± 0.39	1.50 ± 0.27	1.21 ± 0.22
*r*_2_ (mM^−1^s^−1^)	1.27 ± 0.23	320 ± 58	528 ± 95	402 ± 72
*r*_2*_ (mM^−1^s^−1^)	311 ± 47	616 ± 92	542 ± 98	492 ± 74
*r*_2_ */r*_1_	3.18 ± 0.80	148 ± 38	352 ± 90	332 ± 85
*r*_2*_ */r*_1_	779 ± 180	284 ± 66	361 ± 92	407 ± 96

Errors are standard deviation.

**Table 2:** Calculated Spearman correlation coefficients (CC) between the different *LFs* of NF and MF cores for all defined MRI parameters (*RC*, *T*, *R*).

CC	*RC T*_1_ *w*	*RC T*_2_ *w*	*RC T*_2_ *w	*T*_1_	*T*_2_	*T*_2_ *	*R*_1_	*R*_2_	*R*_2*_
NF-MF1	0.48	0.97	0.75	0.67	0.45	0.60	0.67	0.45	0.60
NF-MF2	0.48	0.87	0.73	0.67	0.17	0.62	0.67	0.17	0.62
NF-MF3	0.48	0.95	0.75	0.78	0.72	0.60	0.78	0.72	0.60
MF1-MF2	1.00	0.93	0.98	1.00	0.92	0.98	1.00	0.92	0.98
MF1-MF3	1.00	0.98	1.00	0.97	0.92	1.00	0.97	0.92	1.00
MF2-MF3	1.00	0.95	0.98	0.97	0.75	0.98	0.97	0.75	0.98

## Data Availability

The data presented in this study are available on request from the corresponding author. The data are not publicly available due to institutional restrictions.

## Supplementary Materials

The following are available online at https://www.mdpi.com/article/10.3390/ijms22168487/s1.
