# Supplementary material for: Longitudinal and Transverse Relaxivity Analysis of Native Ferritin and Magnetoferritin at 7 T MRI
Source: Int J Mol Sci. 2021 Aug 6;22(16):8487. doi: 10.3390/ijms22168487 (PMC8395175; doi:10.3390/ijms22168487)
Supplement: Supplementary file 1 [file ijms-22-08487-s001.zip › ijms-1267367-supplementary.pdf]

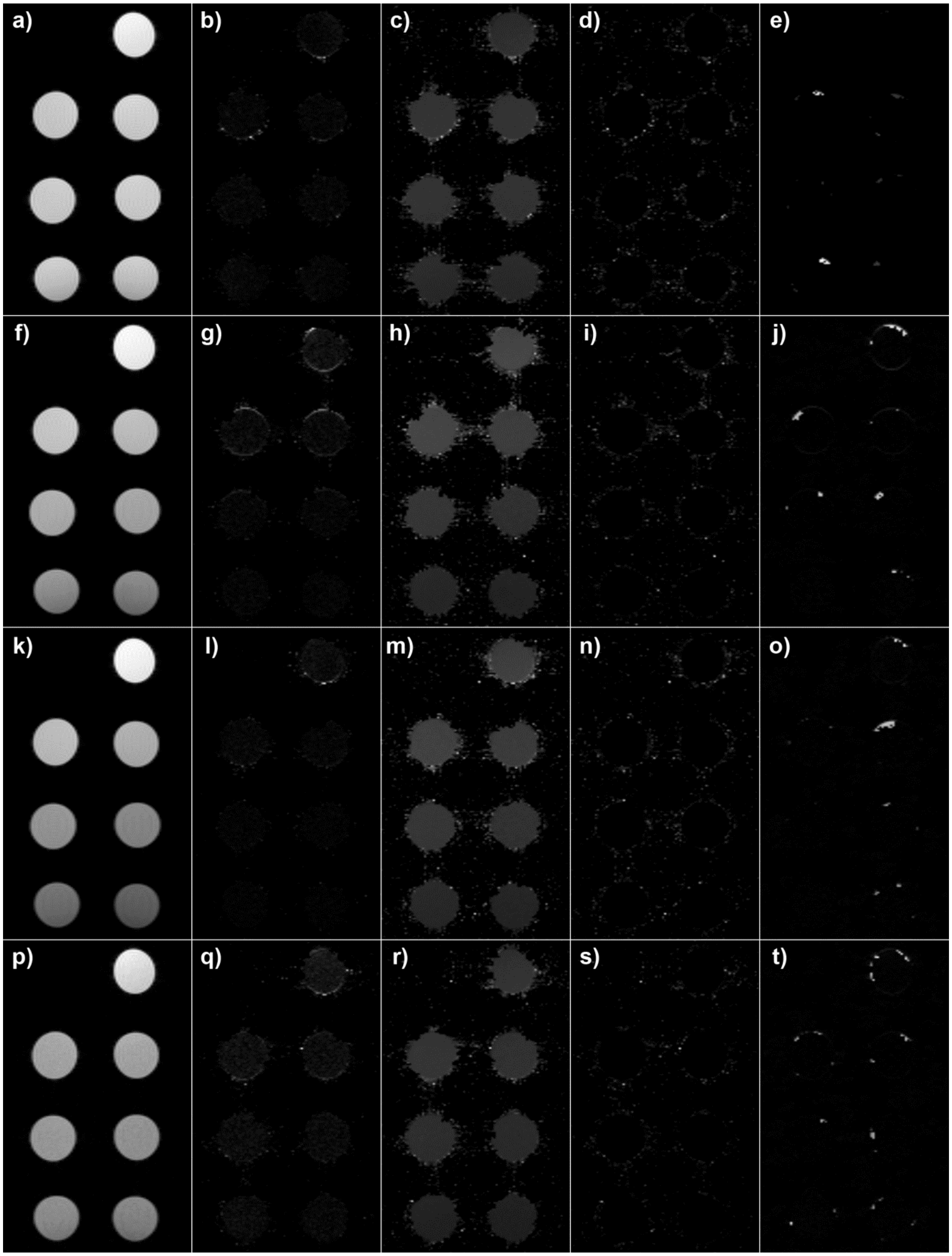

Figure S1. T_1 - weighted image data: (a) NF - signal intensity; (b) NF - standard deviation of signal intensity; (c) NF - relaxation time T_1; (d) NF - standard deviation of relaxation time T_1; (e) NF - standard deviation of the fit; (f) MF1 - signal intensity; (g) MF1 - standard deviation of signal intensity; (h) MF1 - relaxation time T_1; (i) MF1 - standard deviation of relaxation time T_1; (j) MF1 - standard deviation of the fit; (k) MF2 - signal intensity; (l) MF2 - standard deviation of signal intensity; (m) MF2 - relaxation time T_1; (n) MF2 - standard deviation of relaxation time T_1; (o) MF2 - standard deviation of the fit; (p) MF3 - signal intensity; (q) MF3 - standard deviation of signal intensity; (r) MF3 - relaxation time T_1; (s) MF3 - standard deviation of relaxation time T_1; (t) MF3 - standard deviation of the fit.

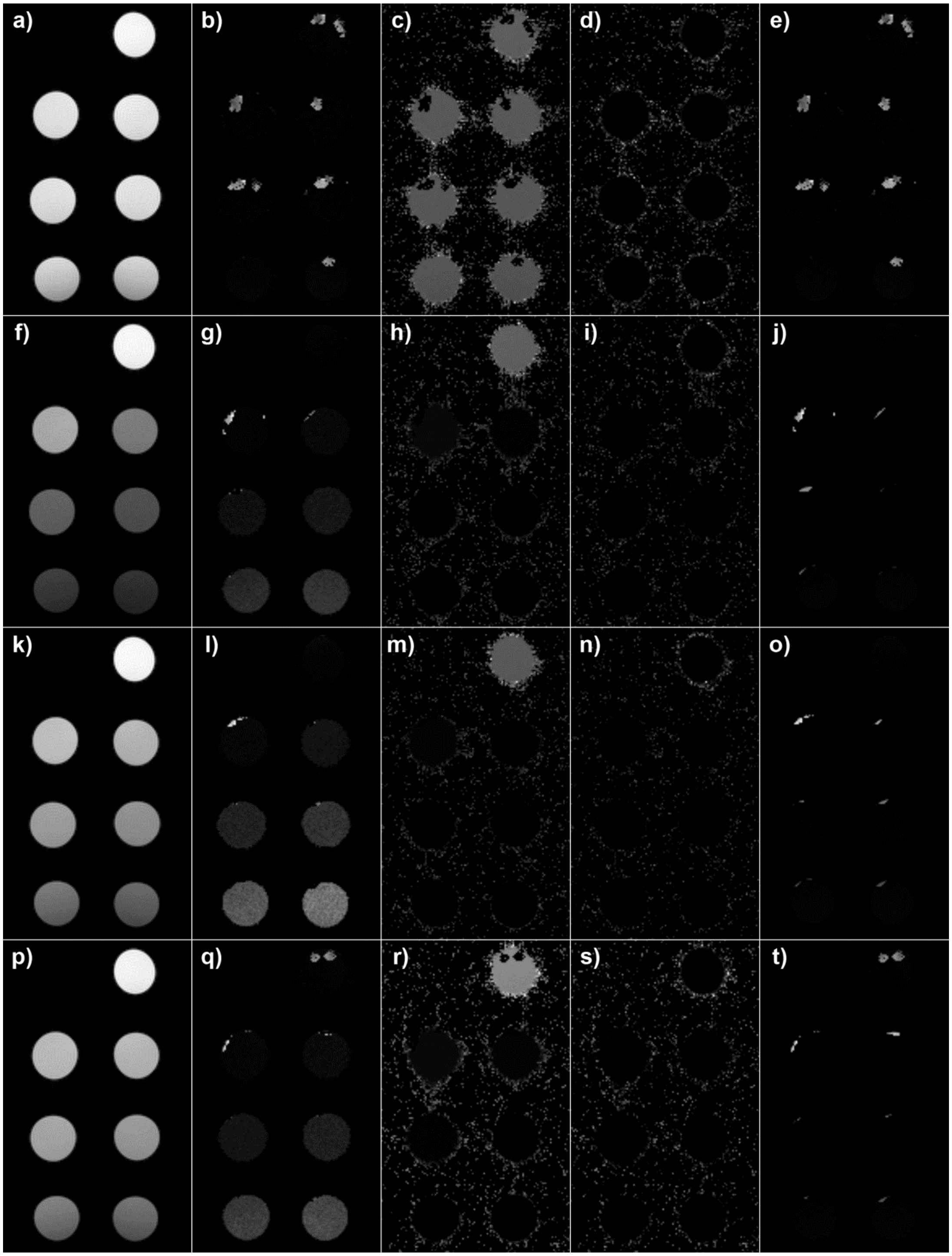

Figure S2. T_2 - weighted image data: (a) NF - signal intensity; (b) NF - standard deviation of signal intensity; (c) NF - relaxation time T_2; (d) NF - standard deviation of relaxation time T_2; (e) NF - standard deviation of the fit; (f) MF1 - signal intensity; (g) MF1 - standard deviation of signal intensity; (h) MF1 - relaxation time T_2; (i) MF1 - standard deviation of relaxation time T_2; (j) MF1 - standard deviation of the fit; (k) MF2 - signal intensity; (l) MF2 - standard deviation of signal intensity; (m) MF2 - relaxation time T_2; (n) MF2 - standard deviation of relaxation time T_2; (o) MF2 - standard deviation of the fit; (p) MF3 - signal intensity; (q) MF3 - standard deviation of signal intensity; (r) MF3 - relaxation time T_2; (s) MF3 - standard deviation of relaxation time T_2; (t) MF3 - standard deviation of the fit.

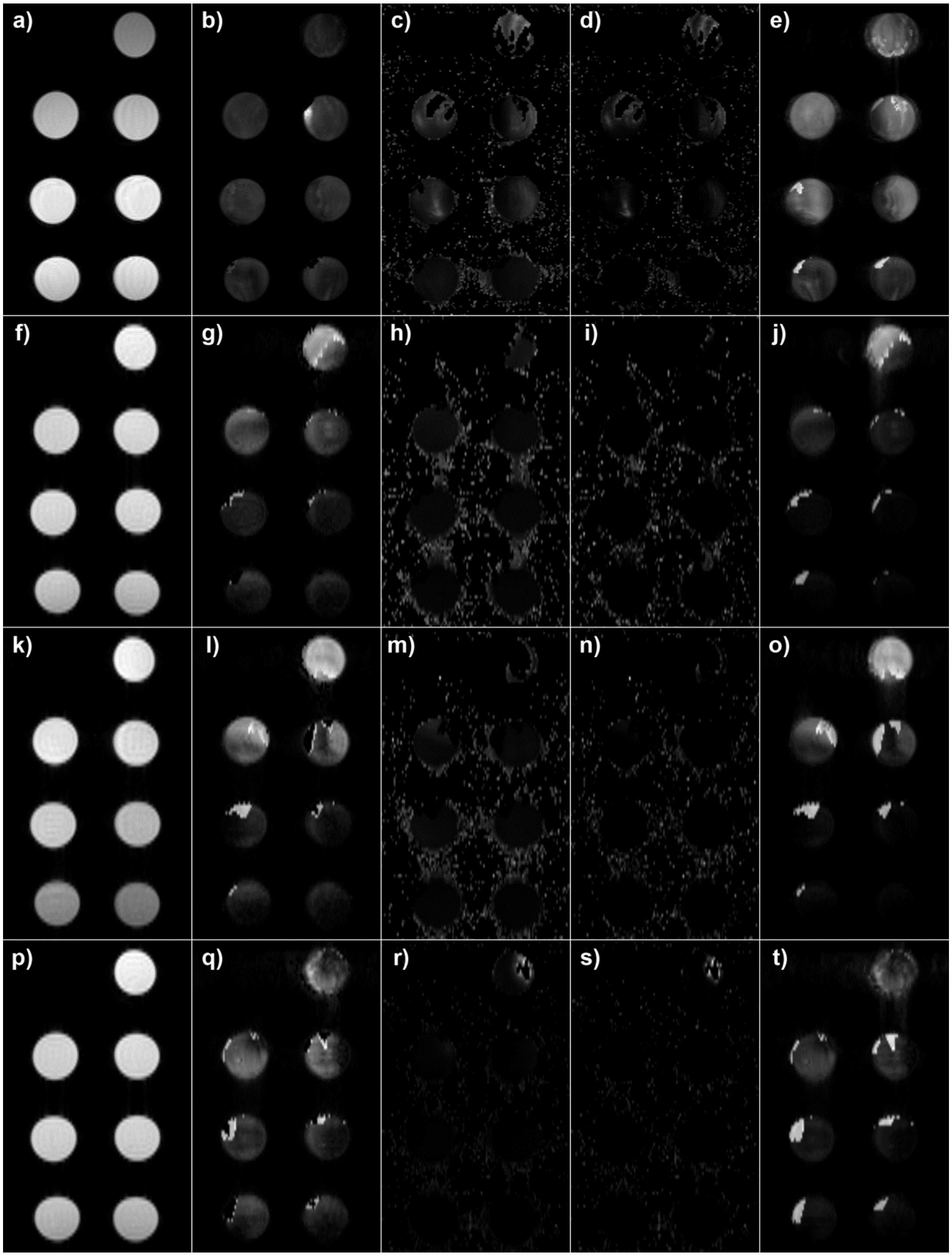

Figure S3. T_2^* - weighted image data: (a) NF - signal intensity; (b) NF - standard deviation of signal intensity; (c) NF - relaxation time T_2^*; (d) NF - standard deviation of relaxation time T_2^*; (e) NF - standard deviation of the fit; (f) MF1 - signal intensity; (g) MF1 - standard deviation of signal intensity; (h) MF1 - relaxation time T_2^*; (i) MF1 - standard deviation of relaxation time T_2^*; (j) MF1 - standard deviation of the fit; (k) MF2 - signal intensity; (l) MF2 - standard deviation of signal intensity; (m) MF2 - relaxation time T_2^*; (n) MF2 - standard deviation of relaxation time T_2^*; (o) MF2 - standard deviation of the fit; (p) MF3 - signal intensity; (q) MF3 - standard deviation of signal intensity; (r) MF3 - relaxation time T_2^*; (s) MF3 - standard deviation of relaxation time T_2^*; (t) MF3 - standard deviation of the fit.

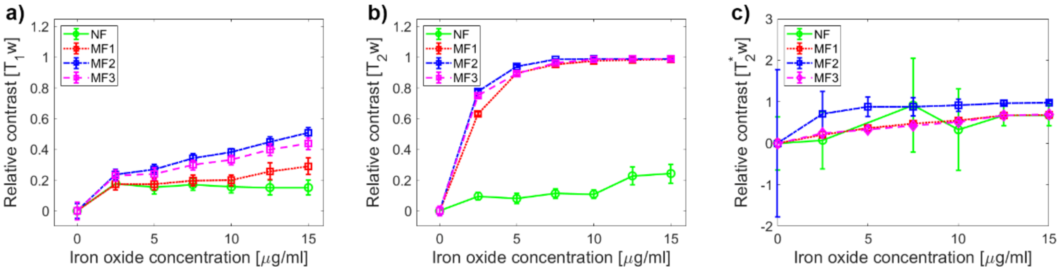

Figure S4. Relative contrast (*RC*) comparison of NF and MF samples: (a) longitudinal relaxation time T_1; (b) transverse relaxation time T_2; (c) transverse relaxation time T_2^*.

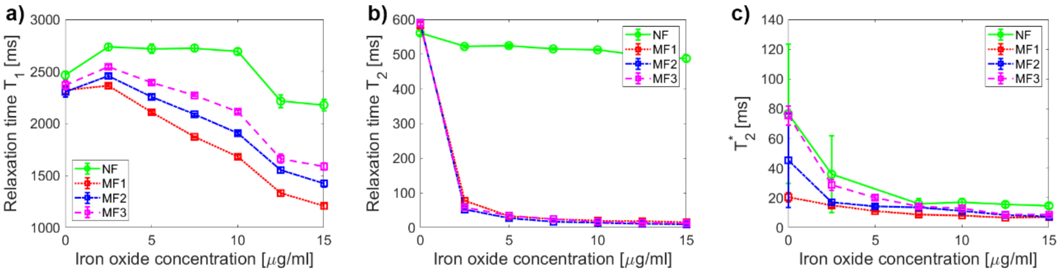

Figure S5. Relaxation times (*T*) comparison of NF and MF samples: (a) longitudinal relaxation time T_1; (b) transverse relaxation time T_2; (c) transverse relaxation time T_2^*.

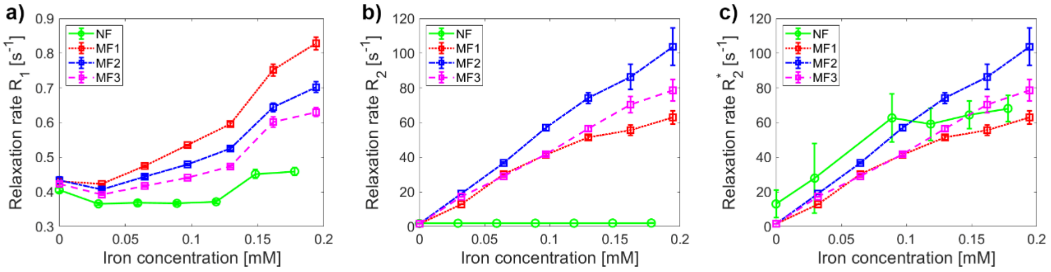

Figure S6. Relaxation rates (*R*) comparison of NF and MF samples: (a) longitudinal relaxation rate R_1; (b) transverse relaxation rate R_2; (c) transverse relaxation rate R_2^*.

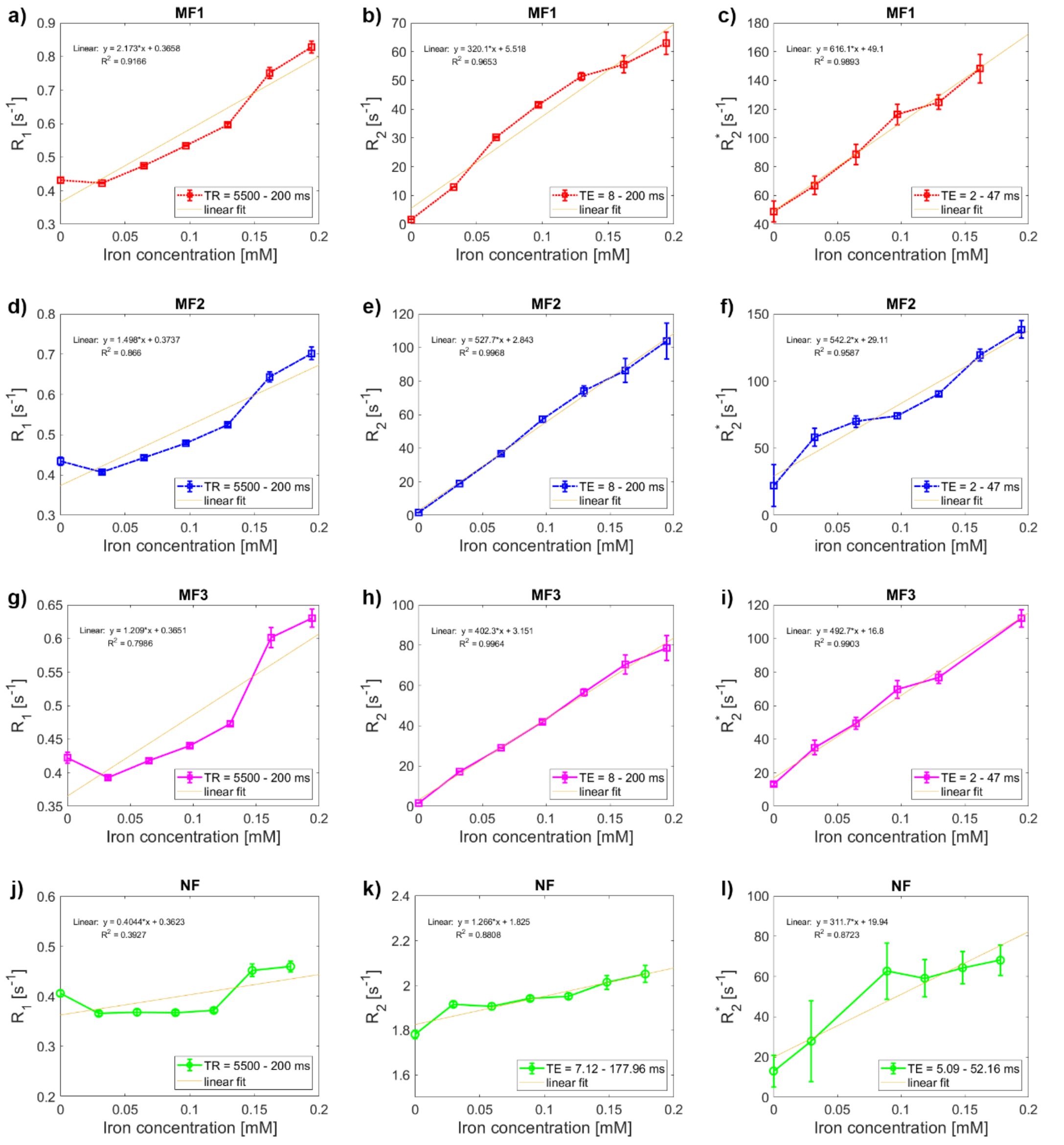

Figure S7. Relaxivity (*r*) determination from the linear fit of relaxation rate (*R*): (a) longitudinal relaxivity r_1 for MF1; (b) transverse~~al~~ relaxivity r_2 for MF1; (c) transverse~~al~~ relaxivity r_2^* for MF1; d) longitudinal relaxivity r_1 for MF2; (e) transverse~~al~~ relaxivity r_2 for MF2; (f) transverse~~al~~ relaxivity r_2^* for MF2; g) longitudinal relaxivity r_1 for MF3; (h) transverse~~al~~ relaxivity r_2 for MF3; (i) transverse~~al~~ relaxivity r_2^* for MF3; j) longitudinal relaxivity r_1 for NF; (k) transverse~~al~~ relaxivity r_2 for NF; (l) transverse~~al~~ relaxivity r_2^* for NF.

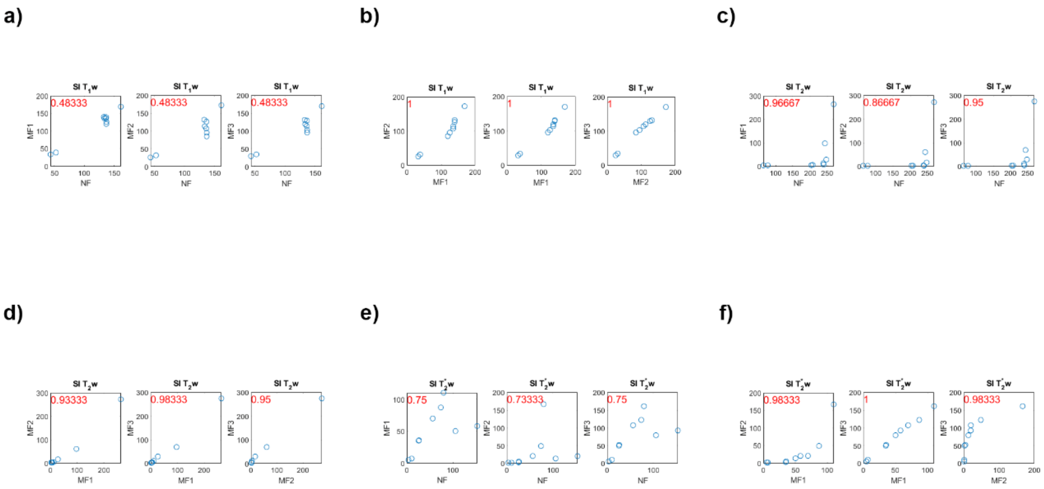

Figure S8. Relative contrast (*RC*)'s correlation coefficients: (a) T_1-weighted NF-MF; (b) T_1-weighted MF-MF; (c) T_2-weighted NF-MF; (d) T_2-weighted MF-MF; (e) T_2^*-weighted NF-MF; (f) T_2^*-weighted MF-MF.

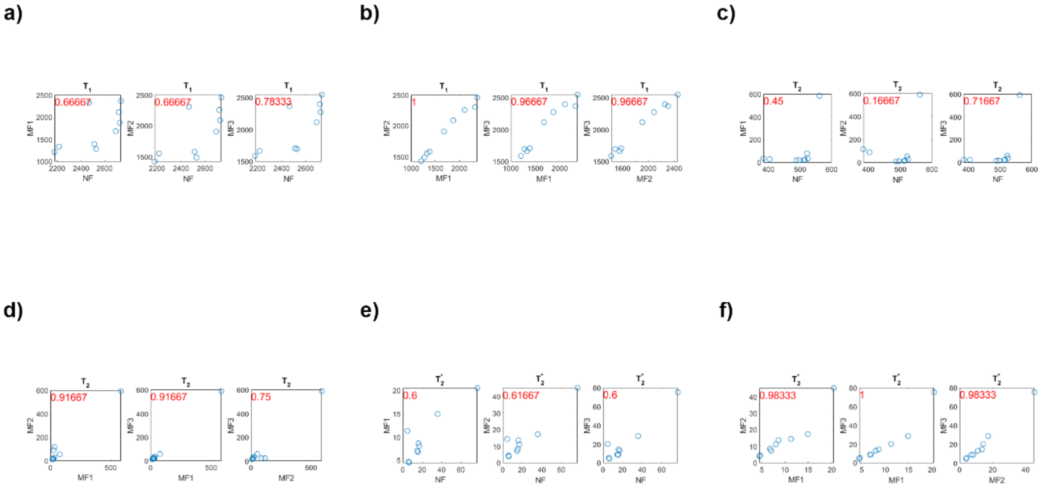

Figure S9. Relaxation time (*T*)'s correlation coefficients: (a) T_1-weighted NF-MF; (b) T_1-weighted MF-MF; (c) T_2-weighted NF-MF; (d) T_2-weighted MF-MF; (e) T_2^*-weighted NF-MF; (f) T_2^*-weighted MF-MF.

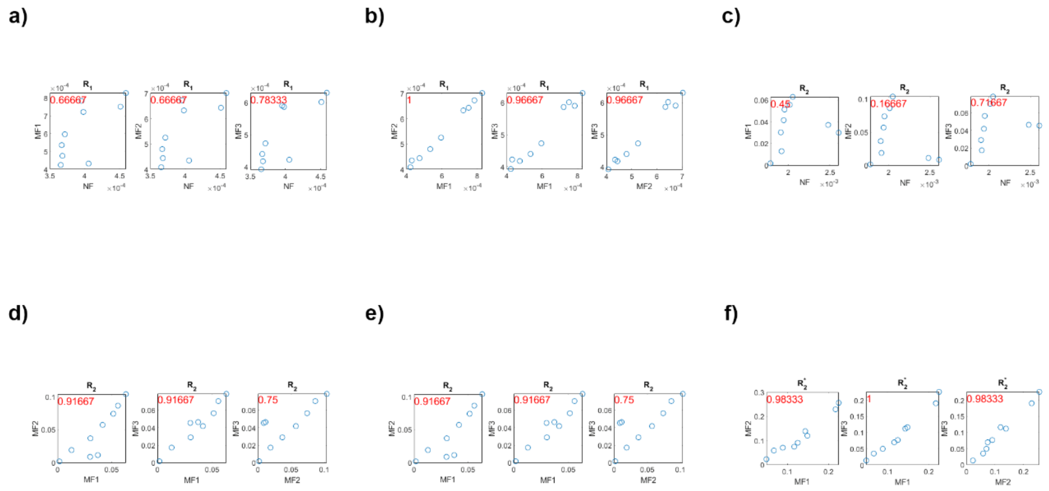

Figure S10. Relaxation rate (R)'s correlation coefficients: (a) T_1-weighted NF-MF; (b) T_1-weighted MF-MF; (c) T_2-weighted NF-MF; (d) T_2-weighted MF-MF; (e) T_2^*-weighted NF-MF; (f) T_2^*-weighted MF-MF.
